# The effect of fingolimod on regulatory T cells in a mouse model of brain ischaemia

**DOI:** 10.1186/s12974-021-02083-5

**Published:** 2021-01-30

**Authors:** Kyle Malone, Andrea C. Diaz Diaz, Jennifer A. Shearer, Anne C. Moore, Christian Waeber

**Affiliations:** 1 Department of Pharmacology and Therapeutics, Western Gateway Building, University College Cork, Cork, Ireland; 2 School of Pharmacy, University College Cork, Cork, Ireland; 3 School of Biochemistry and Cell Biology, University College Cork, Cork, Ireland

**Keywords:** Stroke, Immunomodulation, Ischaemia, Regulatory T cells, Fingolimod, Neuroinflammation

## Abstract

**Background:**

The role of the immune system in stroke is well-recognised. Fingolimod, an immunomodulatory agent licensed for the management of relapsing-remitting multiple sclerosis, has been shown to provide benefit in rodent models of stroke. Its mechanism of action, however, remains unclear. We hypothesised fingolimod increases the number and/or function of regulatory T cells (Treg), a lymphocyte population which promotes stroke recovery. The primary aim of this study was to rigorously investigate the effect of fingolimod on Tregs in a mouse model of brain ischaemia. The effect of fingolimod in mice with common stroke-related comorbidities (ageing and hypercholesteremia) was also investigated.

**Methods:**

Young (15–17 weeks), aged C57BL/6 mice (72–73 weeks), and ApoE^−/−^ mice fed a high-fat diet (20–21 weeks) underwent permanent electrocoagulation of the left middle cerebral artery. Mice received either saline or fingolimod (0.5 mg/kg or 1 mg/kg) at 2, 24, and 48 h post-ischaemia via intraperitoneal injection. Another cohort of young mice (8–9, 17–19 weeks) received short-term (5 days) or long-term (10 days) fingolimod (0.5 mg/kg) treatment. Flow cytometry was used to quantify Tregs in blood, spleen, and lymph nodes. Immunohistochemistry was used to quantify FoxP3+ cell infiltration into the ischaemic brain.

**Results:**

Fingolimod significantly increased the frequency of Tregs within the CD4+ T cell population in blood and spleen post-ischaemia in all three mouse cohorts compared to untreated ischemic mice. The highest splenic Treg frequency in fingolimod-treated mice was observed in ApoE^−/−^ mice (9.32 ± 1.73% vs. 7.8 ± 3.01% in young, 6.09 ± 1.64% in aged mice). The highest circulating Treg frequency was also noted in ApoE^−/−^ mice (8.39 ± 3.26% vs. 5.43 ± 2.74% in young, 4.56 ± 1.60% in aged mice). Fingolimod significantly increased the number of FoxP3+ cells in the infarct core of all mice. The most pronounced effects were seen when mice were treated for 10 days post-ischaemia.

**Conclusions:**

Fingolimod increases Treg frequency in spleen and blood post-ischaemia and enhances the number of FoxP3+ cells in the ischaemic brain. The effect of fingolimod on this regulatory cell population may underlie its neuroprotective activity and could be exploited as part of future stroke therapy.

**Supplementary Information:**

The online version contains supplementary material available at 10.1186/s12974-021-02083-5.

## Background

Over the last few decades, hundreds of agents targeting various pathophysiological mechanisms have undergone pre-clinical testing for the treatment of stroke [1]. Despite limited clinical translation, however, research into neuroprotective molecules in stroke has increased our understanding of the disease [2]. The role of the immune system in all stages of stroke is now being recognised, from the pathogenesis of risk factors to tissue repair [3, 4]. A host of immunomodulatory therapeutic strategies have been suggested as a result [5]. Fingolimod, a sphingosine-1-phosphate receptor agonist first marketed for the treatment of relapsing multiple sclerosis (MS), is one such therapy [6].

Fingolimod has proven effective in various rodent models of stroke, including both transient and permanent focal ischaemia [7]. The drug significantly reduces both infarct volume and neurological deficits, even when administered several hours after the onset of ischaemia [8]. In fact, fingolimod is not only effective in an acute or subacute setting, but also promotes repair in rodents when administered as late as 24 h post-ischaemia [9].With increasing prevalence and effectiveness of reperfusion therapy, and considering that fingolimod is effective against ischemia-reperfusion injury in various organs, the neuroprotective effect of fingolimod could synergize with the effect of increased blood flow to the infarct site [10, 11]. Small-scale pilot studies investigating fingolimod in ischemic stroke patients demonstrated the drug to be safe and effective, including when combined with thrombolysis [12–14]. Nevertheless, while considerable evidence supports the future use of fingolimod in stroke therapy, the exact mechanism of action remains unclear, although immunomodulation is likely to be involved [15].

In general, fingolimod administration post-stroke has been associated with reduced levels of inflammatory markers in the brain, improved endothelial function, and decreased incidence of haemorrhagic transformation [6]. It is widely accepted that fingolimod reduces neuroinflammation in MSby abrogating the sphingosine-1-phosphate (S1P)-dependent egress of naive and central memory T cells from lymph nodes [16]. Pre-clinical stroke studies employing lymphocyte-deficient Rag1^−/−^ mice support the involvement of T cells in fingolimod-afforded neuroprotection [17]. However, in separate studies, fingolimod treatment has failed to significantly reduce infarct volume or neurobehavioral dysfunction despite inducing profound lymphopenia, indicating that other mechanisms may contribute [18].

Regulatory T cells (Tregs) appear to improve disease outcome in stroke, with the only detrimental effects thought to be a role in secondary microthrombosis [19]. Tregs, characterised in mice by high and constitutive expression of the alpha subunit of the IL-2 receptor, CD25, and the transcription factor forkhead box P3 (FoxP3), constitute approximately 20% of all CD4+ T cells found in the ischaemic hemisphere in mice [20]. Mechanisms of Treg-mediated neuroprotection include secretion of IL-10, production of TGF-β and IL-35, priming of M2 macrophages, and reduced inflammatory cytokine secretion [19, 21–24]. While Tregs may contribute to both short-term neuroprotection and long-term stroke recovery, clinical evidence suggests a reduction in circulating number and functional impairment in peripheral blood post-stroke [25]. An imbalance between Th17 cells and Tregs has also been shown to occur [26, 27]. Several recent studies indicate that fingolimod increases the number of Treg and regulatory properties in humans, rodents, and in vitro [28–32]. These observations, combined with the beneficial effects of both fingolimod and Tregs in ischaemic stroke, suggest that fingolimod-mediated neuroprotection may be mediated, at least in part, by Tregs.

The principal aim of this study was to test the hypothesis that fingolimod treatment increases Treg frequency in the blood and secondary lymphoid organs of mice at 1 week post-ischaemia. A second objective was to determine whether fingolimod increases the number of infiltrated FoxP3+ T cells into the infarcted brain. A third objective was to investigate whether the immunomodulatory effects of fingolimod treatment on Treg cells differed in mice with stroke comorbidities, namely aged mice and ApoE^−/−^ mice fed a high-fat diet (the latter representative of patients suffering from hypercholesteremia). Age and hypercholesteremia were chosen as comorbidities due to known aberrations in Treg number and function in these states, alongside the increased stroke risk [33, 34].

A 3-day course of daily fingolimod treatment was administered in this study, in line with clinical trials of the drug in ischaemic stroke [12, 14]. A follow-up study then investigated the effect of short-term (5 days) vs. long-term (10 days) fingolimod administration to test the hypothesis that protracted treatment showed enhanced effects. We used a model of permanent distal middle cerebral artery occlusion (pMCAO) [35]. This model is representative of human middle cerebral artery infarction in which cerebral reperfusion does not occur. Similar to most human stroke cases, the infarct is located principally within the cortex [36]. Moreover, the model provides high reproducibility and low mortality, and has been strongly recommended for studies in which clinical translation is a primary focus [37]. As a result, this study was capable of testing fingolimod in a pre-clinical model of brain ischaemia most closely matched to human stroke.

## Methods

### Ethics statement

Animal experiments were carried out in accordance with the European Directive 2010/63/EU, following approval by the Animal Ethics Committee of University College Cork and under an authorization issued by the Health Products Regulatory Authority Ireland (license number AE19130/P042 and AE19130/P075). The study was conducted and its immune-related results reported according to the ARRIVE guidelines [38]. Results of histological and functional stroke outcome measures will be reported elsewhere and the link to the current study appropriately acknowledged.

### Mice

All mice were sourced from Envigo unless otherwise stated. For experiments carried out in designated “young” mice populations, male C57BL/6 (15–17 weeks old) mice were used unless otherwise stated. For experiments carried out in “aged” mice, C57BL/6 male and female mice were obtained at 58–62 weeks of age (Charles River, UK); procedures were carried out at 72–73 weeks of age. Male ApoE^−/−^ mice were received at 4–5 weeks of age and starting at 8 weeks of age were placed on an adjusted calorie high fat diet (TD.88137) for 12 weeks. Subsequent experiments were carried out at 20–21 weeks of age. All mice were acclimatised for at least 1 week before any procedure took place. Mice were group housed in individually ventilated cages in a specific-pathogen-free facility. Mice were exposed to a 12-h light/12-h dark cycle and kept at a temperature of 20–24 **°**C and a relative humidity of 45–65%. Mice were provided with environmental enrichment and ad libitum access to both food and water. A total of *n* = 257 mice were included as part of this study. Data from an initial pilot study confirmed *n* > 3 per group would be sufficient to detect the expected effect of fingolimod on Treg frequency in spleen, while *n* > 6 per group would be sufficient to detect the expected change of CD3+ cells in the brain. Notably, for all experiments, *n* = 16 mice were allocated to each group unless otherwise stated. This number was determined by an a priori sample size calculation conducted as part of a larger research project investigating the effect of fingolimod on histological and functional stroke outcomes. Based on a previously published meta-analysis [39], 16 mice per group were required to detect changes in infarct size and neurological score (significance level set at α = 0.05, power of 80%). Although we confirmed *n* = 6 mice per group should be sufficient to detect changes in peripheral Treg frequencies and brain cell counts, where possible, tissue from all mice under study was collected to both maximize power for other measurements and to allow for comparison of immunological results with other outcomes. Pre-determined exclusion criteria included mice with serious uncontrollable haemorrhage as well as mice with thermal or physical damage of the cortex. The number of mice excluded for meeting such criteria or humane endpoints are outlined for each cohort in Additional file 5.

### Ischaemia Model

A permanent distal middle cerebral artery occlusion model was employed, as previously described [35]. All mice were randomly allocated to treatment groups using an online pseudo-random number generator (randomizer.org) a few days prior to surgery. Mice were anaesthetised by vaporiser with isoflurane (3–4% for induction; 1–2% for maintenance) in O_2_/N_2_ (30%/70%)_._ Suitable depth of anaesthesia was confirmed by absence of the pedal withdrawal reflex. Body temperature, monitored by rectal probe, was maintained at 37 °C with a homeothermic blanket system. Hair removal cream (Veet) was applied to the area between the left ear and left eye. The site was washed repeatedly with 70% alcohol and iodine. The site was anesthetised topically with 0.5% bupivacaine (0.1 ml) before being incised. The temporal muscle was retracted to expose the temporal and parietal bones. In sham animals, only the skin incision and exposure of the skull was performed. A small craniotomy was performed over the bifurcation of the middle cerebral artery (MCA) and the dura was removed to expose the MCA (including parietal and frontal branches). The distal portion of the MCA, including the branches and the main artery below the bifurcation, were occluded using small vessel bipolar electrocoagulation forceps (Bovie Bantam Pro electrosurgical generator (A952) /McPherson 3 1/2” straight forceps (A842) (Symmetry Surgical Inc., USA)). The MCA was nicked to confirm successful occlusion of the artery and prevent reperfusion. The burr hole was then covered with bone wax and the incision sutured. Anaesthesia was discontinued and mice were allowed to recover in a heated chamber (32 °C) for 30 min before being returned to their home cage. Recovery of experimental mice was monitored daily through use of a scoresheet which graded weight loss, appearance changes, behaviour, and neurological function. At 2, 24, and 48 h post-ischaemia, young mice received either normal saline or 0.5 mg/kg or 1 mg/kg fingolimod (Novartis Institutes for Biomedical Research, Basel) via i.p. injection. Aged and ApoE^−/−^ mice received either normal saline or 0.5 mg/kg fingolimod via i.p. injection. For young mice allocated to the treatment duration study, mice received either normal saline or fingolimod (0.5 mg/kg) for 5 days followed by normal saline or fingolimod (0.5 mg/kg) for 10 days. A researcher not associated with the surgery prepared treatment solutions (pH 7) for volumes no greater than 250 μl per injection.

### Tissue collection and processing

At 7 days post-ischaemia (or at 10 days for the treatment duration study), mice were culled by anaesthetic overdose (Euthatal, 200 mg/ml i.p.; Merial). Approximately 500 μl of blood was collected from the descending aorta and transferred into EDTA-coated tubes. Mice were then perfused transcardially with 20 ml cold phosphate-buffered saline (PBS). The brain was removed from the skull, frozen in isopentane (- 42 °C), and stored at – 20 °C. Brain sections (20 μm) were cut at 500 μm intervals on a cryostat and stored at – 20 °C. Cervical lymph nodes, inguinal lymph nodes, and spleen tissue were harvested and stored in PBS until further manipulation. Spleen and lymph node tissues were mechanically dissociated using the back of a plunger of a 3 ml syringe in approximately 3 ml PBS in a sterile 6-well plate. The resulting cell suspensions were passed through a 70-μm cell strainer and collected in a 50-mL conical tube. Wells and strainers were washed twice with 1X Dulbecco’s PBS (Sigma-Aldrich, #D8537). Spleen and blood samples were re-suspended in 5 mL of 1× Red Blood Cell Lysis Buffer and incubated for 5 min at room temperature (eBioscience, #430054). The lysis reaction was stopped by adding 20 mL of 1X PBS. All samples were then washed twice with 1× PBS, re-suspended in an appropriate volume of PBS and counted using trypan blue to determine total cell concentration and viability.

### Flow cytometric analysis

**Fig. 1:**
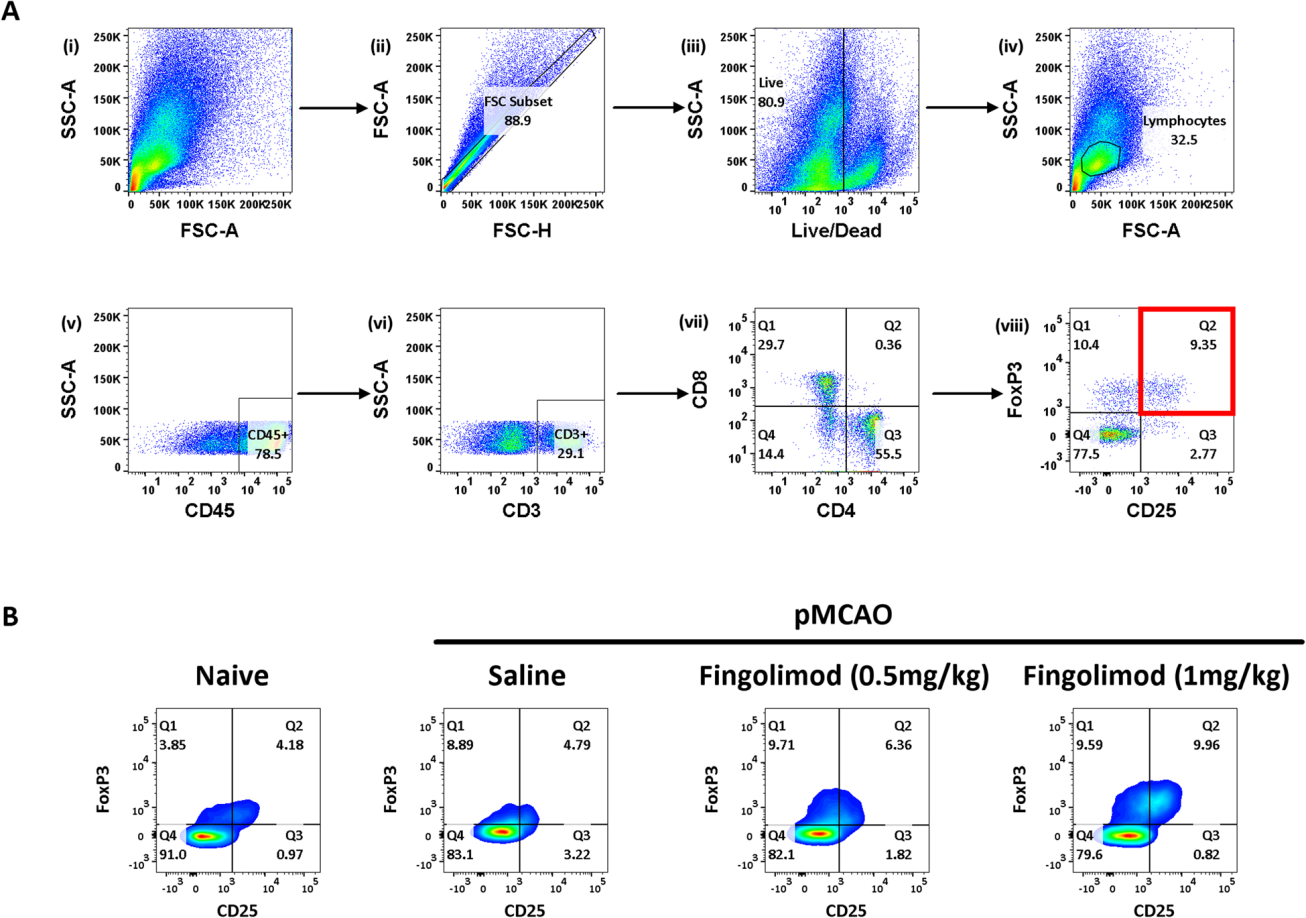
**a** Gating strategy for determination of Treg frequency (i) = initial population, (ii) = singlets, (iii) = live cells, (iv) = lymphocytes, (v) = CD45hi cells, (vi) = CD3+ cells (T cells), (vii) = CD4+ T cells vs. CD8+ T cells, (viii) = CD4+ CD25+ FoxP3+ cells (quadrant in red) designated Tregs. **b** Flow cytometric analysis of splenic Treg frequencies in a mouse model of brain ischaemia. 15–17-week-old male, C57BL/6 mice underwent permanent middle cerebral artery occlusion (pMCAO). At 2, 24, and 48 h post-ischaemia, mice received either saline or fingolimod (0.5 mg/kg or 1 mg/kg) by intraperitoneal injection (*n* = 16 mice per group). Age-matched mice who had not undergone pMCAO acted as immunological controls (*n* = 8). At 7 days post-ischaemia, flow cytometry was used to determine the frequency of splenic Treg cells. All gates were determined by both negative cells and fluorescence minus one controls

Samples were incubated for 5 min with 50 μl of anti-mouse CD16/CD32 (Clone 93, 1:100) (eBioscience, #14016182). The respective cell suspensions were then stained for anti-mouse CD45 (PerCP-CY5.5, # 45045182) (30-F11, 1:100), CD3 (PE-Cy7, #14003182) (145-2C11, 1:100), CD4 (FITC, #11004282) (RM4-5, 1:800), CD8 (Pacific Blue, #MCD0828) (5H10, 1:100), and CD25 (APC, #17025182) (PC61.5, 1:100) (eBioscience). A live/dead stain (1:10,000 solution) was also added to each sample (Fixable Viability Dye eFluor 780, #65086514 eBioscience). Samples were then incubated for 30 min at 2–8 °C in the dark. Post-incubation, the samples were washed, fixed, permeabilised, and stained intracellularly for anti-mouse FoxP3 (1:100, FJK-16s, # 12577382), in accordance with the instructions provided with the Mouse Regulatory T Cell Staining Kit #1 (eBioscience, # 88811140). Samples were then re-suspended in an appropriate volume of PBS. Flow cytometric analysis was performed with a LSRII flow cytometer (Becton Dickinson). Compensation control was set using BD CompBead Anti-Rat/Anti-Hamster Particles Set (#552845). Data was analysed using Flowjo (v10) according to the following gating strategy: live cells (as determined by live/dead stain), lymphocytes (as determined by FSC/SSC), T lymphocytes (as determined by CD3+), CD4+ and CD8+, and regulatory T cells (CD4+ cells which co-express CD25 and FoxP3). Gates were set according to unstained samples and fluorescent minus one controls. The gating strategy required for the determination of Treg frequency (expressed as CD4+ CD25+ FoxP3+/CD4+ %) is outlined in Fig. 1. Absolute cell counts for all tissues were calculated in accordance with the instructions provided with the CountBright Absolute Counting Beads (Molecular Probes, # C36950). All results are reported according to the Minimum Information About a Flow Cytometry Experiment (MIFlowCyt) [40].

### Immunohistochemistry

Immunohistochemical analysis of immune cell infiltration into brain tissue was performed on *n* = 10 mice per group. Slides were brought from – 20 °C to room temperature and dried overnight. Sections were fixed with ice cold acetone (– 20 °C) for 10 min, dried for 30 min at room temperature and washed twice with PBS. Slides were incubated in blocking solution (5% Rabbit Serum (Vector Laboratories, # S-5000) in 0.01M PBS) for 30 min and endogenous biotin was blocked according to the instructions provided with the Avidin/Biotin Blocking Kit (Vector Laboratories, #SP-2001). Sections were incubated with primary antibodies against either CD3 (145-2C11, 1:500, #14003182) or FoxP3 (FJK-16s, 1:300, #14577382) at room temperature for 60 min (eBioscience). Slides were washed twice with PBS then incubated with 3% H_2_O_2_ (Sigma, #H1009) for 30 min. Subsequently, slides were incubated with either a biotinylated goat anti-hamster (1:500, # PA132045) or a donkey anti-rat secondary antibody (1:500, #A18739) for 60 min at room temperature (eBioscience). Slides were washed twice with PBS. Immunoreactivity was visualized by the avidin-biotin complex method (Vectastain avidin-biotin complex Kit, #PK-4000, Vector Laboratories) and developed for 10 min with diaminobenzidine (DAB, Vector Laboratories, #SK-4100). Slides were counterstained with eosin and then dehydrated sequentially in graded ethanol (70% for 2 min, 95% for 2 min, 100% for 2 min). Slides were washed twice in HistoChoice (Sigma, #H2779) for 2 min and then mounted with Permount (Fischer Scientific, #SP15-500). Finally, slides were visualized under an Olympus BX51 microscope (Additional file 1). A single representative image of the ischaemic core, undamaged ipsilateral tissue, and contralateral tissue were taken at the × 10 objective lens. Three random representative images of the peri-infarct zone were also taken at this magnification. Positively stained cells were counted in the relevant regions of interest using ImageJ [41].

### Statistical analysis

The Kolmogorov–Smirnov test was used to test normality of flow cytometric data, which are displayed as 10–90 percentile box-and-whisker plots. The ROUT method (*Q* = 1%) was used to identify possible outliers, which were then screened for exclusion. Two-sided, independent-samples*t* tests were employed to investigate differences between two groups. One-way analysis of variance (ANOVA) followed by post hoc Tukey’s multiple comparisons was used to investigate differences between three or more groups. Two-way analysis of variance (ANOVA) followed by post hoc Tukey’s multiple comparisons was used to investigate differences between three or more groups when the effect of two independent variables were studied. A *p* value < 0.05 was considered statistically significant. Statistical analysis was performed using GraphPad Prism 8.0. All investigators remained blinded to treatment groups throughout analysis.

## Results

### Fingolimod increases Treg frequency in murine spleen and blood post-pMCAO

The impact of fingolimod on peripheral Tregs (defined as CD4+ CD25+ FoxP3+ cells) was first established via flow cytometric analysis of blood, spleen, draining lymph nodes (cervical), and non-draining lymph nodes (inguinal) harvested from young, male mice at 7 days post-ischaemia. Mice received either normal saline, 0.5 mg/kg fingolimod, or 1 mg/kg fingolimod via i.p. injection at 2 hours post-pMCAO and again 24 and 48 h later. Age-matched mice (*n* = 8) that had not undergone either surgery or treatment acted as naïve controls. 

In this initial dose optimisation study, the expected lymphopenia associated with fingolimod treatment was confirmed (see Additional file 2). As Tregs make up one portion of the total CD4+ cell compartment, the known effect of fingolimod on CD4+ cell counts was also replicated [42, 43]. In particular, a reduction in CD4+ counts in spleen and blood was elucidated (see Additional file 3).

**Fig. 2:**
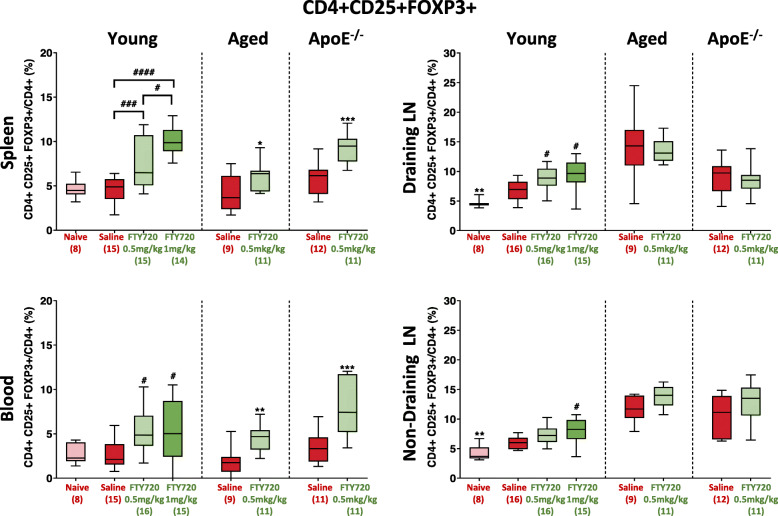
Frequency of CD4+ CD25+ FoxP3+ cells in the blood and secondary lymphoid tissue across three individual mouse cohorts of brain ischaemia (*t* = 7 days) as a result of fingolimod (FTY720) treatment. Two-sided, independent-samples *t* tests investigated differences between two groups (* = *p* < 0.05, ** = *p*<0.01, *** = *p* < 0.001 as compared to saline). One-way analysis of variance (ANOVA) tests with post hoc Tukey’s multiple comparisons were performed to investigate differences between three or more groups (# = *p* < 0.05, ## = *p* < 0.01, ### = *p* < 0.001 as compared to saline). Number of mice per group is shown in parentheses on *x*-axis. Box-and-whisker plots exhibit 10–90 percentiles

In comparison to non-ischaemic controls, stroke caused a significant increase in Treg frequency in both draining (*p* = 0.0080) and non-draining (*p* = 0.0019) lymph nodes (Fig. 2). No significant differences in Treg frequency were observed between ischaemic and non-ischaemic mice in spleen or blood.

Fingolimod significantly increased Treg frequency in the spleens of young mice post-ischaemia in a dose-dependent manner (*p* < 0.0001). The 0.5 mg/kg dose was used for experiments in aged and ApoE^−/−^ mice. 

In aged and ApoE^−/−^ mice (all male), fingolimod (0.5 mg/kg) significantly increased splenic Treg frequency (*p* = 0.0348, 0.0002, respectively), as per young mice. Likewise, this dose of fingolimod was sufficient to increase Treg frequency in blood in all populations under study (young *p* = 0.0195, aged *p* = 0.0011, ApoE^−/−^ mice *p* = 0.0003). In draining lymph nodes, fingolimod (0.5 mg/kg) only boosted Treg frequency in young mice (*p* = 0.0140). In non-draining lymph nodes, only the higher dose (1 mg/kg) produced a significant response (*p* = 0.0150). Once more, however, this increase was only recorded in young mice.

Overall, these results demonstrate that fingolimod treatment significantly increases the frequency of CD4+CD25+FoxP3+ Treg cells in the periphery (spleens and blood) of all three cohorts under study. Significant increases in Treg frequency in lymph nodes were only observed in young mice.

### Age has a major impact on peripheral Treg frequencies in lymph nodes at baseline and at 7 days post-pMCAO

In the last few years, the pivotal role draining lymph nodes in particular may play in brain immune surveillance and the development of neuroinflammatory disorders has been proposed [44]. Given the profound differences observed in Treg frequency in draining lymph nodes between young and aged mice post-pMCAO in Fig. 2, a follow-up study investigated whether age, ischaemia, or a combination of both factors was responsible. Peripheral Treg frequency was established in young (*n* = 7 males, 6 females) and aged (*n* = 6 males, 8 females) mice cohorts at 7 days post-ischaemia. A corresponding group of young (*n* = 7 males, 7 females) and aged (*n* = 7 males, 7 females) untreated naïve mice acted as non-ischaemic controls. A separate group of young mice (*n* = 9) underwent sham surgery as detailed above to confirm the validity of using non-ischaemic naïve mice as immunological controls. 

**Fig. 3:**
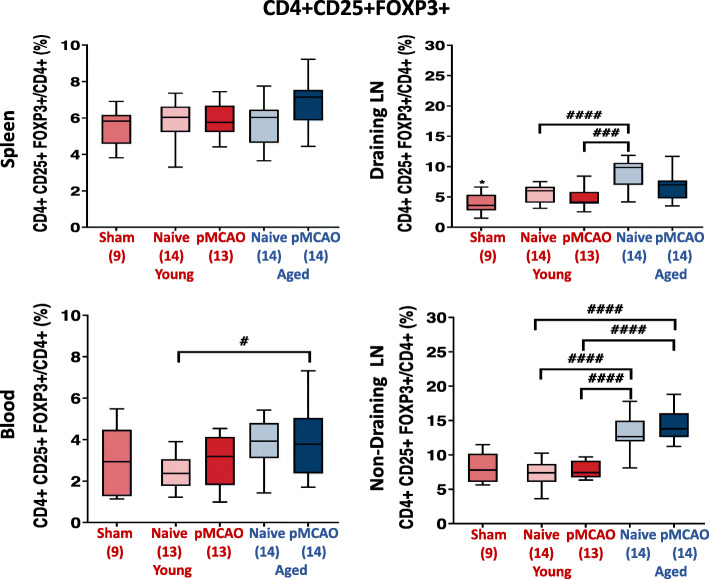
Effect of pMCAO on the frequency of CD4+ CD25+ FoxP3+ cells in blood and secondary lymphoid tissue of young and aged mice (*t* = 7 days). Young mice are shown in red, aged mice are shown in blue. Sham mice only received initial incision and exposure of the skull. Naïve mice were age-matched mice who did not undergo any surgery. Two-sided, independent-samples *t* tests investigated differences between two groups (* = *p* < 0.05, ** = *p* < 0.01, *** = *p* < 0.001. Two-way analysis of variance (ANOVA) tests with post hoc Tukey’s multiple comparisons were performed to investigate differences between three or more groups (# = *p* < 0.05, ## = *p* < 0.01, ### = *p* < 0.001). The number of mice per group is shown in parentheses on *x*-axis. Box-and-whisker plots exhibit 10–90 percentiles

When comparing sham mice to untreated young naïve mice, a significant difference in Treg frequency was only noted in draining lymph nodes (*p* = 0.0362). Treg frequencies in spleen and blood were not affected by the induction of ischaemia in either young or aged mice (Fig. 3). Similarly, no change was observed in cervical or inguinal lymph nodes. This latter result conflicts with results seen in the first group of young mice (Fig. 2), where ischaemia increased Treg frequency in lymph nodes. It should be noted that the supplier for young mice differed between these particular studies (Envigo, UK (Fig. 2) vs. Charles River, UK (Fig. 3)). It has been recognised that slight changes in T cell phenotype may be observed between strains [45]. In blood, higher Treg frequencies were noted in aged mice (*p* = 0.0039). Likewise, in draining lymph nodes and non-draining lymph nodes, significantly higher Treg frequencies were observed in naïve aged mice compared to naïve young mice (*p* < 0.0001). This demonstrates that age was the major factor which determined differences in peripheral Treg frequencies between groups, whereas ischaemia tended to reduce Treg frequency only in draining lymph nodes *(p* = 0.0271) but not in other sites. The sex of mice did not influence Treg frequency (Additional file 4). Separately, no immunological controls for ApoE^−/−^ mice fed a high-fat diet were available, meaning the effect of ischaemia on peripheral Treg frequencies in this cohort remains unknown.

### Extending fingolimod treatment duration from 5 days to 10 days post-pMCAO increased Treg frequencies in lymph nodes

**Fig. 4:**
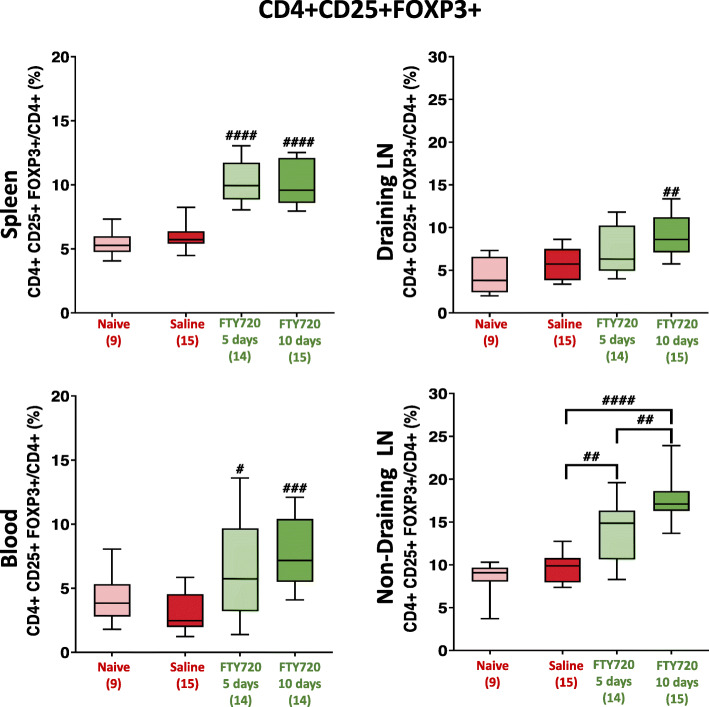
Frequency of CD4+ CD25+ FoxP3+ cells in blood and secondary lymphoid tissue in response to short-term and long-term fingolimod (FTY720) treatment post-brain ischaemia in young mice (*t* = 10 days). Two-sided, independent-samples *t* tests investigated differences between two groups (* = *p* < 0.05, ** = *p* < 0.01, *** = *p* < 0.001 as compared to saline). One-way analysis of variance (ANOVA) tests with post hoc Tukey’s multiple comparisons were performed to investigate differences between three or more groups (# = *p* < 0.05, ## = *p* < 0.01, ### = *p* < 0.001 as compared to saline). Number of mice per group is shown in parentheses on *x*-axis. Box-and-whisker plots exhibit 10–90 percentiles

To date, the effect of fingolimod treatment has not been reported beyond 7 days post-ischaemia in a pre-clinical study [7]. However, evidence from MS patients treated with fingolimod suggests the amplification of Tregs may increase with longer-term administration [42, 46]. As a result, the effect of an extended treatment duration on peripheral Treg frequencies at 10 days post-pMCAO in young, male mice was also investigated (Fig. 4).

 Extending drug treatment from 5 to 10 days did not further increase the frequency of Tregs in circulation (spleen and blood). However, in lymph nodes, extended fingolimod treatment had a significant impact on the frequency of Tregs. In draining lymph nodes, 10-day fingolimod treatment significantly increased Treg frequency (*p* = 0.0028), whereas 5-day treatment only resulted in a trend towards increased Treg frequency. In non-draining lymph nodes, however, both short-term (*p* = 0.0016) and long-term (*p* = 0.0062) fingolimod treatment significantly enhanced Treg frequency. Overall, longer-term fingolimod treatment increased Treg frequency in all peripheral tissues analysed. Notably, short-term fingolimod treatment only failed to enhance Treg frequency in draining lymph nodes, suggesting many immunomodulatory effects of the drug remain for at least 5 days after withdrawal.

### Fingolimod increases FoxP3+ cell infiltration into the brain post-pMCAO

**Fig. 5:**
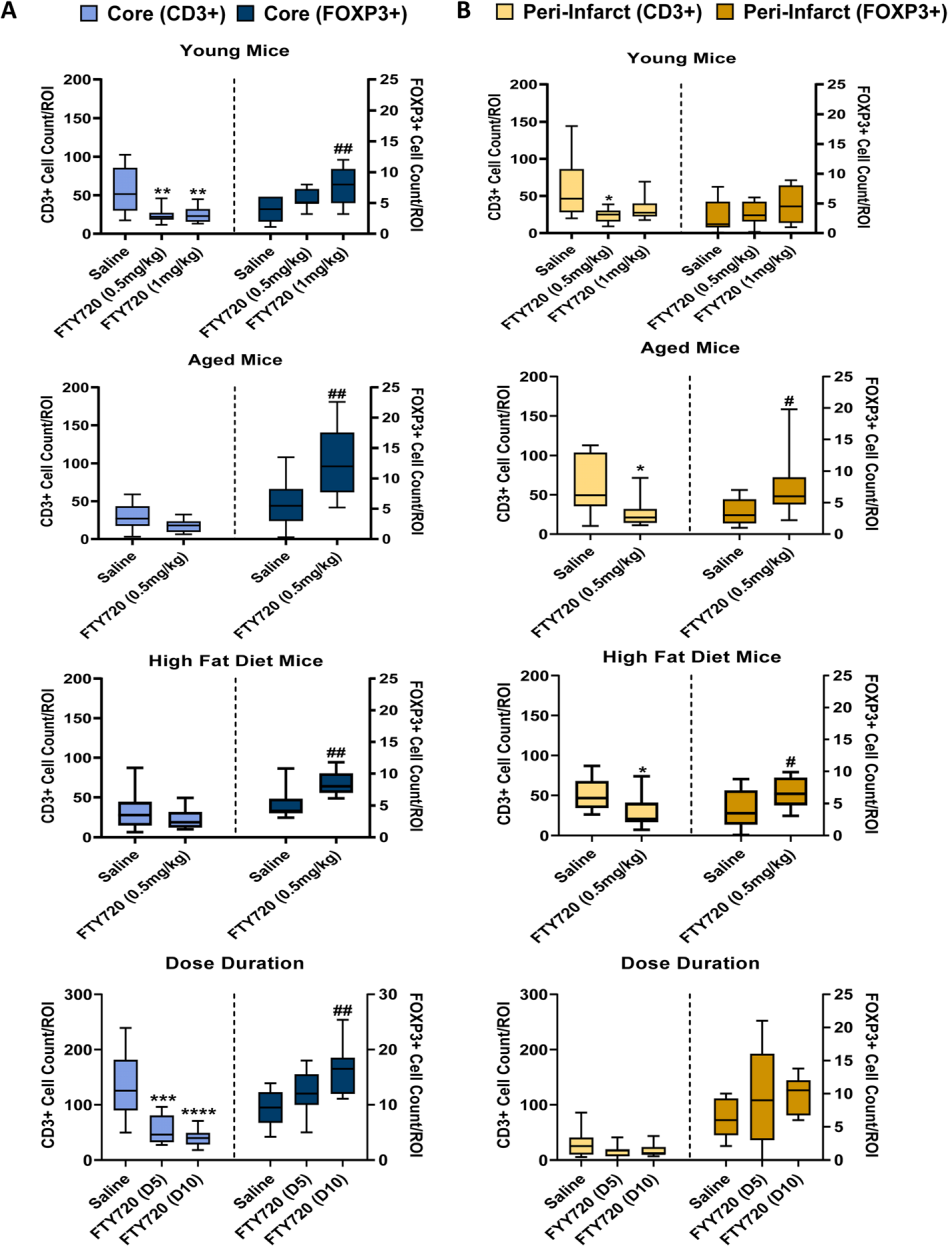
Total CD3+ and FoxP3+ cell counts in the infarct core (**a**) and peri-infarct zone (**b**) of mice post-pMCAO (*t* = 7–10 days) (*n* = 10 per group). DAB-based immunohistochemistry was used to determine cell counts. Two-sided, independent-samples *t* tests investigated differences between two groups. One-way analysis of variance (ANOVA) tests with post hoc Tukey’s multiple comparisons were performed to investigate differences between three or more groups. For comparisons between CD3+ groups, * = *p* < 0.05, ** = *p* < 0.01, *** = *p* < 0.001 (vs. saline). For comparison between FoxP3+ groups, # = *p* < 0.05, ## = *p* < 0.01, ### = *p* < 0.001 (vs. saline)

The effect of fingolimod on Treg numbers in the brain post-ischaemia was investigated by immunohistochemical labelling of brain sections (*n* = 10 per group) with anti-CD3 and anti-FoxP3 antibodies. Positively stained cells were counted in both the ischaemic core (Fig. 5a) and peri-infarct (Fig. 5b) regions, in accordance with the results of previous studies [47, 48].

In young mice, both doses of fingolimod reduced the total number of CD3+ T cells infiltrating into the ischaemic core at 7 days post-pMCAO (*p* = 0.0048 and 0.0060, respectively). Low dose fingolimod (0.5 mg/kg) also reduced CD3+ cell counts in the peri-infarct region (*p* = 0.0153). Interestingly, the administration of fingolimod caused an increase in the number of FoxP3+ cells in the core, which was significant in the high dose group of young mice (*p* = 0.0016). No effect of fingolimod on FoxP3+ cell numbers was observed in the peri-infarct of young mice. In aged mice, low dose fingolimod did not significantly reduce total CD3+ T cell numbers in the ischaemic core at 7 days post-pMCAO (*p* = 0.0825), but a significant decrease in the peri-infarct region was observed (*p* = 0.0192). Significant increases in FoxP3+ cell counts were noted in both the core and peri-infarct regions of aged mice treated with fingolimod (*p* = 0.0035 and 0.0437 respectively). Fingolimod had a similar effect in ApoE^−/−^ mice fed a high-fat diet. Specifically, low dose fingolimod failed to produce any significant change in CD3+ T cells in the ischaemic core (*p* = 0.3475) but reduced CD3+ numbers in the peri-infarct region (*p* = 0.0492). Likewise, significantly higher numbers of FoxP3+ cells were observed in both the core (*p* = 0.0073) and peri-infarct region (*p* = 0.0492).

In order to investigate the potential relationship between fingolimod treatment duration and FoxP3+ cell infiltration into the brain post-pMCAO, a final study with low dose fingolimod was conducted. Low dose fingolimod significantly decreased total T cell numbers in the core in both short-term (*p* = 0.0005) and long-term treatment groups (*p* < 0.0001), whereas only long-term fingolimod dosing increased FoxP3 cells in the ischaemic core at 10 days post-pMCAO (*p* = 0.0019). Overall, these results highlight that fingolimod treatment results in significant enrichment of FoxP3+ cells in the infarct site, independently of age or comorbidity. In young mice, fingolimod also significantly depletes CD3+ cells at the infarct site.

## Discussion

Fingolimod, approved for the treatment of MS, shows promise as a clinical candidate for stroke treatment [49]. Despite supporting data from numerous pre-clinical studies, questions related to the mechanism(s) of action persist. This study used immunohistochemical and flow cytometric analysis to explore the effect of fingolimod on the known neuroprotective lymphocyte subpopulation of regulatory T cells in a mouse model of brain ischaemia. Overall, our results demonstrate that fingolimod treatment significantly increased Treg frequency in the spleen and blood of young, aged, and ApoE^−/−^ mice at 7 days post-brain ischaemia. Fingolimod significantly increased Treg frequency in both draining and non-draining lymph nodes of young but not aged or ApoE^−/−^ mice post-ischaemia. Finally, fingolimod enhanced the number of FoxP3+ cells in the infarcted brain, independently of age or comorbidity.

The relative strengths of our study include appropriate sample sizes determined by a priori power calculation, the administration of two different fingolimod doses, and the testing of numerous dosing schedules. The use of mice with common stroke-related comorbidities also increases the clinical relevance of this work [50]. Conversely, a limitation of our study is that the Treg profile was only examined at a single time-point in each study. Previous research was consulted to choose a time-point at which Treg entry to the brain was expected [51]. Nevertheless, the precise relationship between onset of therapy and increased Tregs in our model was not examined. Furthermore, the impact of fingolimod treatment on the Treg suppressive function in the setting of ischaemic stroke was not determined. Finally, only the electrocoagulation model of permanent middle cerebral artery occlusion was employed in this study. Notably, a previous published study demonstrated that fingolimod failed to improve experimental stroke outcome in young mice in this model [18]. Conversely, the drug has proven effective in mice which underwent transient models of middle cerebral artery occlusion (tMCAO) [7]. Some evidence suggests a cerebral ischemia–reperfusion model such as tMCAO should be preferentially used for testing anti-inflammatory drugs [52]. This is particularly true in cases where the drug candidate may be used as an adjunct to thrombolysis [53].

Previous studies in MS, acute kidney injury, and colitis showed that fingolimod increased the proportion of Tregs in blood and secondary lymphoid tissues [43, 54–57]. In stroke, other Treg-modulating therapies, including CD28 superagonist, IL-33, IL-2/IL-2-antibody complex, and adoptive Treg cell transfer, have been shown to increase peripheral Treg frequencies by two- to three-fold in young mice [58–61]. However, questions related to these modulators remain, including issues of cost, length of treatment window, potential toxicity of Treg stimulants, and the stability of the expanded Tregs for these modulators remain [62]. In contrast, an abundance of clinical data exists for the use of fingolimod in central nervous system (CNS) disorders [63]. Therefore, fingolimod may provide a safe and robust alternative for effectively boosting Tregs in the periphery post-stroke. Its known neuro- and vaso-protective properties may also offer added bonuses over other small molecule Treg-modulators such as rapamycin [22].

The mechanism underlying the effect of fingolimod on Tregs could be related to an effect on CD4+ T cells that do not display a regulatory phenotype, i.e. “non-Tregs”. The observation that fingolimod reduced total CD4+ numbers in spleen and blood while increasing Treg frequency suggests Tregs may be refractory to fingolimod treatment. Notably, non-Treg cells have been shown to depend more on the S1P gradient to recirculate from the lymph node to the blood in comparison to Tregs, as evidenced by higher levels of S1PR1 on these cells [64]. Therefore, the modulation of S1PR1 by fingolimod treatment may preferentially sequester non-Tregs in lymph nodes. Alternatively, or additionally, interruption of S1PR1 signalling by fingolimod could enhance modulation of T cells towards a regulatory phenotype. Under normal circumstances, the S1P-mTOR axis antagonises TGF-β function [65, 66]. In the presence of fingolimod, however, internalisation of the S1PR1 may promote expansion of the Treg pool through increased TGF-β-Smad3 signalling. Overall, a combination of reduced non-Treg trafficking and enhanced TGF-β-driven Treg evolution may underlie the observed increased Treg frequencies in young mice post-ischaemia.

In the current study, the effect of fingolimod on Tregs in mice with common stroke comorbidities was also determined. The fingolimod-induced increase in Treg frequency in the spleen and blood of aged mice is of particular interest in light of recent work that highlights the frequency of circulating Tregs correlates with age in ischaemic stroke patients [67]. The magnitude of Treg frequency change in spleen and blood was also notably higher in aged mice than in young mice, suggesting older cohorts may, in theory, derive greater benefit from fingolimod treatment. Separately, the ability of fingolimod to boost Tregs in spleen and blood of ApoE^−/−^ mice fed a high-fat diet is promising, as there is substantial evidence to suggest Treg numbers and function are impaired during atherosclerosis development [68, 69]. Atherosclerosis drives Treg plasticity, resulting in the accumulation of an intermediate dysfunctional Th1-like IFNγ+ phenotype which permits further arterial inflammation [70]. Our results demonstrate that fingolimod may modulate CD4+ T cell populations away from these inflammatory phenotypes towards Tregs post-pMCAO; an effect that, if properly exploited, might improve long-term brain tissue repair [71].

Fingolimod treatment also increased Treg frequency in draining and non-draining lymph nodes in young mice. These results are similar to those observed in experimental autoimmune uveitis [72]. Recently, the pivotal contribution of cervical lymph nodes in particular to post-stroke systemic inflammation and subsequent infarct growth has been described with respect to macrophage-mediated inflammation [73]. The same study suggested T cells may also be involved. Combined with the known ability of lymph node Tregs to influence inflammatory outcomes in distal organs, these findings suggest that in the immediate aftermath of stroke, cervical lymph node Tregs might help coordinate the peripheral immune response through the release of anti-inflammatory cytokines such as IL-10 [74, 75]. In the current study, fingolimod increased Treg frequency in CNS-draining lymph nodes, although the effect was only observed in young mice. Higher Treg frequencies were observed in the lymph nodes of naïve aged compared to naïve young mice (Fig. 3). This is in line with previous results which suggest Tregs accumulate in secondary lymphoid tissue in aged mice [33]. It is possible an upper plateau exists in aged mice beyond which higher Treg frequencies cannot be induced. Therefore, if the primary mechanism of action by which fingolimod affords neuroprotection post-stroke is by increasing Tregs in draining lymph nodes, it would not provide any clinical benefit in older populations. In contrast, the phenotype of lymph node T cells in young mice appears more dynamic and responsive to pharmacological modulation. Previous work examining the chronic accumulation of Tregs in both brain and non-draining lymph nodes post-ischaemia in young mice suggests such long-lasting immunological consequences could positively impact stroke recovery [76]. In particular, the ability of cervical lymph node Tregs to prevent deleterious antigen-specific immune reactions developing post-stroke may play a crucial role [77].

Recent evidence suggests Tregs must traffic to the infarcted brain in order to provide maximum benefit, while effector T cells contribute most to post-stroke neurodegeneration after gaining access to the lesion site [78, 79]. As a result, the crucial balance between regulatory and effector T cells in the post-ischaemic brain was examined by immunohistochemistry. In line with previous results, fingolimod reduced CD3+ cell infiltration in the brains of young mice [17, 18]. Recent research suggests this might be primordially due to interference with S1P receptor signalling which facilitates recruitment of splenic T cells to the infarcted hemisphere [80]. FoxP3+ cells, meanwhile, represented approximately 10% of all infiltrating T cells in saline-treated young mice, in broad agreement with other studies [19]. Uniquely, the FoxP3+ to CD3+ cell ratio was higher in fingolimod-treated aged brains than fingolimod-treated young brains, correlating with results noted in spleen and blood. However, the observation that fingolimod increases FoxP3+ cell counts in the ischaemic brain conflicts with a recent finding wherein once daily dosing of fingolimod reduced brain Tregs [48]. Importantly, however, this same study only initiated fingolimod treatment in the subacute period (6–13 days post-stroke), in direct contrast with the acute dosing regimen chosen here. Furthermore, the study employed a transient (60 min) model of middle cerebral artery occlusion. There are profound differences in the numbers of inflammatory cells which enter the brain when reperfusion is allowed to occur compared to the permanent model utilized here [81]. The higher numbers of leukocytes which infiltrate in the first 24 h after permanent ischaemia may present fingolimod with an increased number of T cells it can direct towards a regulatory phenotype.

Overall, the enhanced number of FoxP3+ cells observed in the infarcted brain as a result of fingolimod treatment may represent increased Treg recruitment or an in situ expansion of the regulatory phenotype in response to the local inflammatory milieu. Chemokine receptors including CCR5, CCR6, and CCR8 have been proposed as mediators of Treg mobilization to the ischaemic brain [79]. The ability of fingolimod to positively influence the capacity of Tregs to traffic to the brain by upregulating expression of these receptors remains unclear [82]. Recent evidence does suggest a link between the drug and the local inflammatory milieu through upregulation of the anti-inflammatory “M2” microglia phenotype [83]. It is possible fingolimod enhances modulation to a Treg phenotype via M2 microglia-mediated release of anti-inflammatory cytokines at the infarct site [84].

The most pronounced effects of fingolimod on brain FoxP3+ cell counts were observed when the drug was administered once daily for 10 days. To date, clinical trials of fingolimod in ischaemic stroke have only administered the drug in the first 72 h post-ischaemia [12–14]. In terms of the future use of extended treatment regimens, careful consideration should be paid to the impact of fingolimod on wider host immunity, especially in the acute post-stroke period where widespread stroke-induced immunodepression occurs [85]. Evidence suggests Tregs might attenuate SIID in the subacute phase after brain injury in mice [86]. Notably, fingolimod does not increase the risk of spontaneous bacterial infection post-cerebral ischaemia in young mice [87]. Therefore, fingolimod may reduce the incidence of bacterial infection through fine-tuning the Treg response in the acute post-stroke period. Nevertheless, the potential for expanded Tregs to convert into pro-inflammatory effector T cells post-ischaemia needs to be balanced with the beneficial effect on neuroinflammation [88].

## Conclusion

Fingolimod has attracted attention in stroke research due to its proposed ability to sequester neurotoxic immune cells within lymph nodes while possibly also providing direct protection to the blood-brain barrier (BBB) and brain cells. This study shows for the first time that fingolimod also increases peripheral Treg frequency post-ischaemia and enhances FoxP3+ cells in the infarcted brain. These effects were observed in young mice but also notably in aged and ApoE^−/−^ mice, two common stroke comorbidities with known alterations in T cell function [70, 89]. The fingolimod dose and treatment schedule needed to maximize these effects were also investigated. While the 0.5 mg/kg dose was shown to increase peripheral and brain Tregs across all models, it is at the lower end of effective doses used in preclinical studies of fingolimod in stroke. Therefore, a longer treatment window may in fact be desirable to ensure improved stroke recovery in the clinic. In order to confirm Tregs play a role in the neuroprotective effect of fingolimod, the drug should now be tested in mice in which Tregs are depleted (e.g. anti-CD25) or downregulated (e.g. diabetic stroke) [51, 90]. Separately, an evaluation of the factors which determine Treg numbers and function in human stroke could help identify further patient populations who may benefit. Finally, the possibility to combine immunomodulatory drugs such as fingolimod with intravenous or intra-arterial reperfusion therapy should be considered, whether as a means to extend the reperfusion window, ameliorate reperfusion injury, or enhance immunomodulatory activity at the infarct site [91]. Overall, this study provides key information on the potential mechanism of action of fingolimod as a treatment for stroke needed for successful clinical translation.

## Supplementary Information

**Additional file 1.** : Representative immunohistochemistry images of CD3+ (A-C) and FoxP3+ (D-F) staining in the infarct core of young mice post-pMCAO (t = 7 days). CD3+ images acquired with the 20X objective lens of an Olympus BX51 microscope. FoxP3+ images acquired with the 40X objective lens. Positive cells counted using ImageJ. A = CD3 (saline), B = CD3 (fingolimod 0.5mg/kg), C = CD3 (fingolimod 1mg/kg). D = FoxP3 (saline), E = FoxP3 (fingolimod 0.5mg/kg), F = FoxP3 (fingolimod 1mg/kg). Sample positive staining indicated via black arrows.

**Additional file 2.** : Total circulating CD3+ cells across three individual mouse cohorts of brain ischaemia (t = 7 days). Two-sided, independent-samples *t* tests investigated differences between two groups (* = p<0.05, ** = p<0.01, *** = p<0.001 as compared to saline). One-way analysis of variance (ANOVA) tests with post hoc Tukey’s multiple comparisons were performed to investigate differences between three or more groups (# = p<0.05, ## = p<0.01, ### = p<0.001 as compared to saline). Number of mice per group shown in parentheses on x-axis.Box-and-whisker plots exhibit 10-90 percentiles.

**Additional file 3.** : Total CD4+ cell counts in blood and secondary lymphoid tissue across three individual mouse cohorts of brain ischaemia (t = 7 days). Two-sided, independent-samples *t* tests investigated differences between two groups (* = p<0.05, ** = p<0.01, *** = p<0.001 as compared to saline). One-way analysis of variance (ANOVA) tests with post hoc Tukey’s multiple comparisons were performed to investigate differences between three or more groups (# = p<0.05, ## = p<0.01, ### = p<0.001 as compared to saline). Number of mice per group shown in parentheses on x-axis.Box-and-whisker plots exhibit 10-90 percentiles.

**Additional file 4.** : Effect of pMCAO on the frequency of CD4+ CD25+ FoxP3+ cells in blood and secondary lymphoid tissue of young (male + female) and aged (male + female) mice (t = 7 days). Young mice are shown in red, aged mice are shown in blue. Male mice are shown in clear boxes, female mice are shown in hatched boxes. Naïve mice were age-matched mice who did not undergo any surgery. One-way analysis of variance (ANOVA) tests with post hoc Tukey’s multiple comparisons were performed to investigate differences between groups (# = p<0.05, ## = p<0.01, ### = p<0.001). The number of mice per group shown in parentheses on x-axis.Box-and-whisker plots exhibit 10-90 percentiles.

**Additional file 5.** : Detailed breakdown of number of mice excluded pre-surgery, mid-surgery for serious uncontrollable haemorrhage, or post-surgery for reaching a humane endpoint. Details of any naïve mice added, as well as samples missed or excluded post-analysis by ROUT method are also outlined.

## Data Availability

The data that supports the findings of this study is available from the corresponding author upon reasonable request and will be stored in a data repository.

## Abbreviations

APOE: Apolipoprotein E
BBB: Blood-brain barrier
FoxP3: Forkhead box protein 3
MCA: Middle cerebral artery
pMCAO: Permanent middle cerebral artery occlusion
ROUT: Robust regression and outlier removal
TGF-β: Transforming growth factor beta
