# Supplementary figures and images for: The effect of fingolimod on regulatory T cells in a mouse model of brain ischaemia
Source: J Neuroinflammation. 2021 Jan 30;18:37. doi: 10.1186/s12974-021-02083-5 (PMC7847573; doi:10.1186/s12974-021-02083-5)

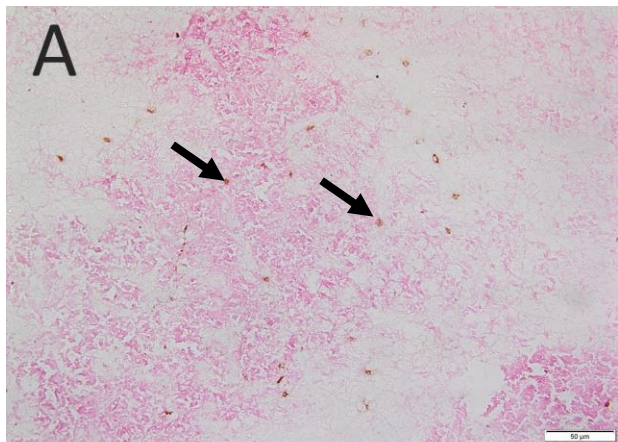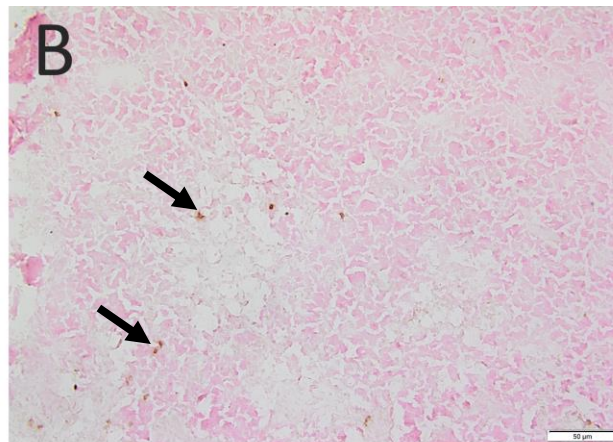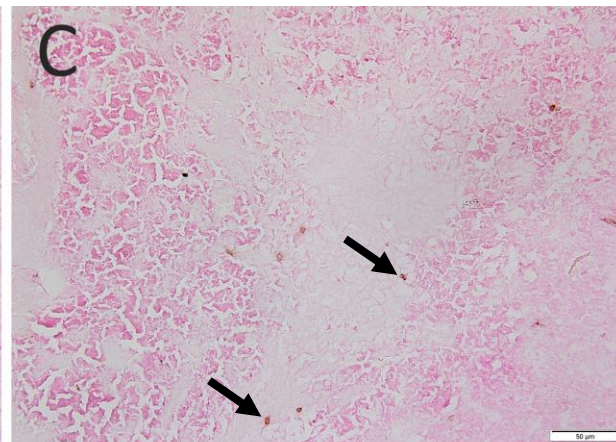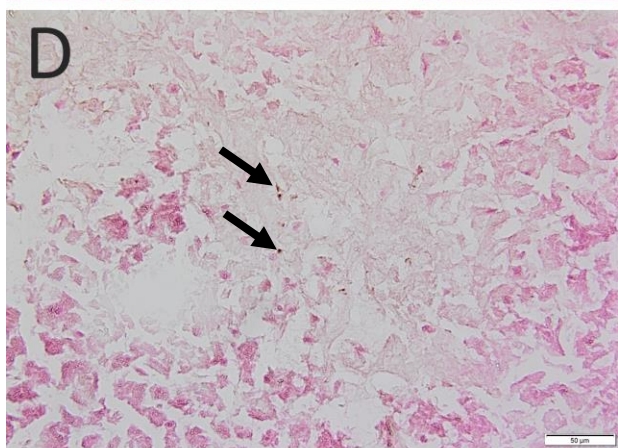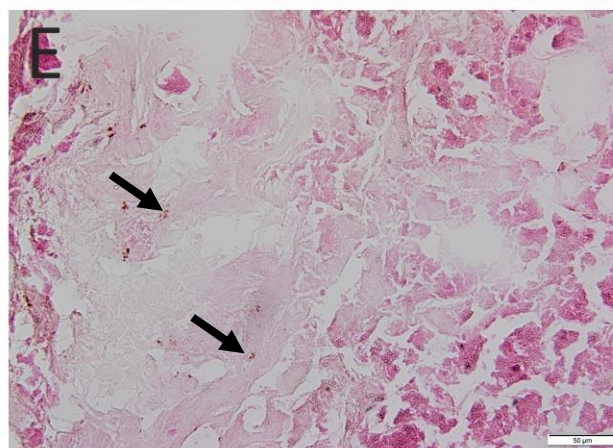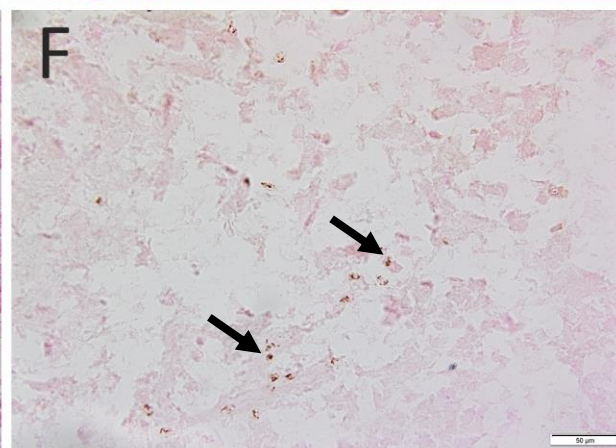

Supplement: Supplementary file 1 — Additional file 1. : Representative immunohistochemistry images of CD3+ (A-C) and FoxP3+ (D-F) staining in the infarct core of young mice post-pMCAO (t = 7 days). CD3+ images acquired with the 20X objective lens of an Olympus BX51 microscope. FoxP3+ images acquired with the 40X objective lens. Positive cells counted using ImageJ. A = CD3 (saline), B = CD3 (fingolimod 0.5mg/kg), C = CD3 (fingolimod 1mg/kg). D = FoxP3 (saline), E = FoxP3 (fingolimod 0.5mg/kg), F = FoxP3 (fingolimod 1mg/kg). Sample positive staining indicated via black arrows. [file 12974_2021_2083_MOESM1_ESM.pdf]

## CD3+ Cell Count

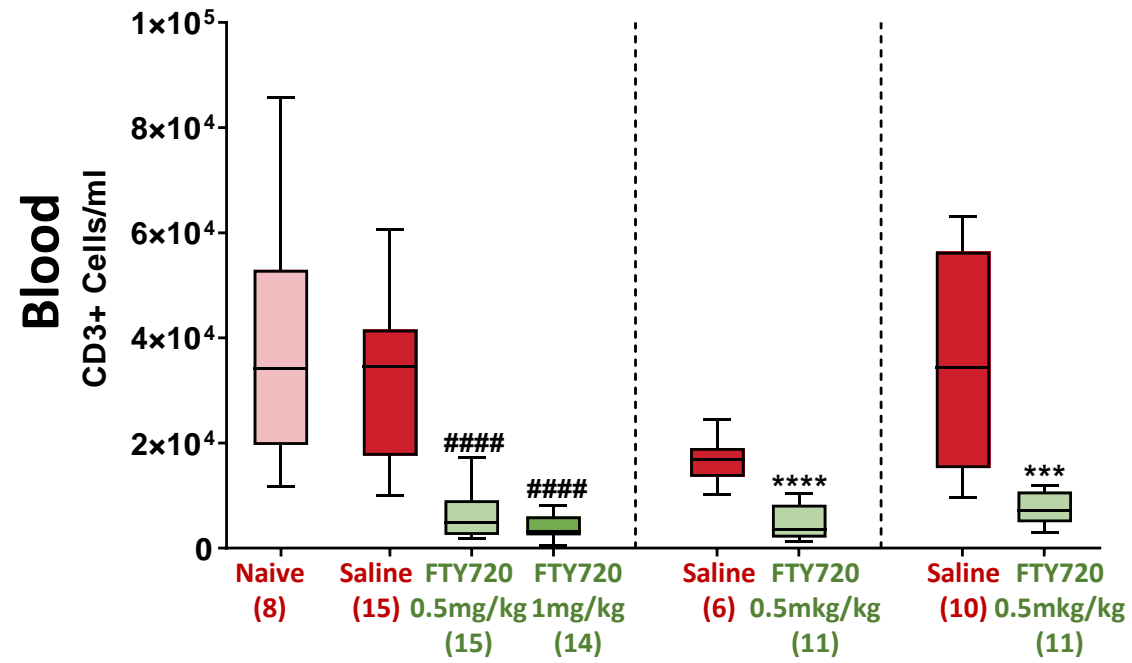

Supplement: Supplementary file 2 — Additional file 2. : Total circulating CD3+ cells across three individual mouse cohorts of brain ischaemia (t = 7 days). Two-sided, independent-samples t tests investigated differences between two groups (* = p<0.05, ** = p<0.01, *** = p<0.001 as compared to saline). One-way analysis of variance (ANOVA) tests with post hoc Tukey’s multiple comparisons were performed to investigate differences between three or more groups (# = p<0.05, ## = p<0.01, ### = p<0.001 as compared to saline). Number of mice per group shown in parentheses on x-axis.Box-and-whisker plots exhibit 10-90 percentiles. [file 12974_2021_2083_MOESM2_ESM.pdf]

CD4+ Cell Count

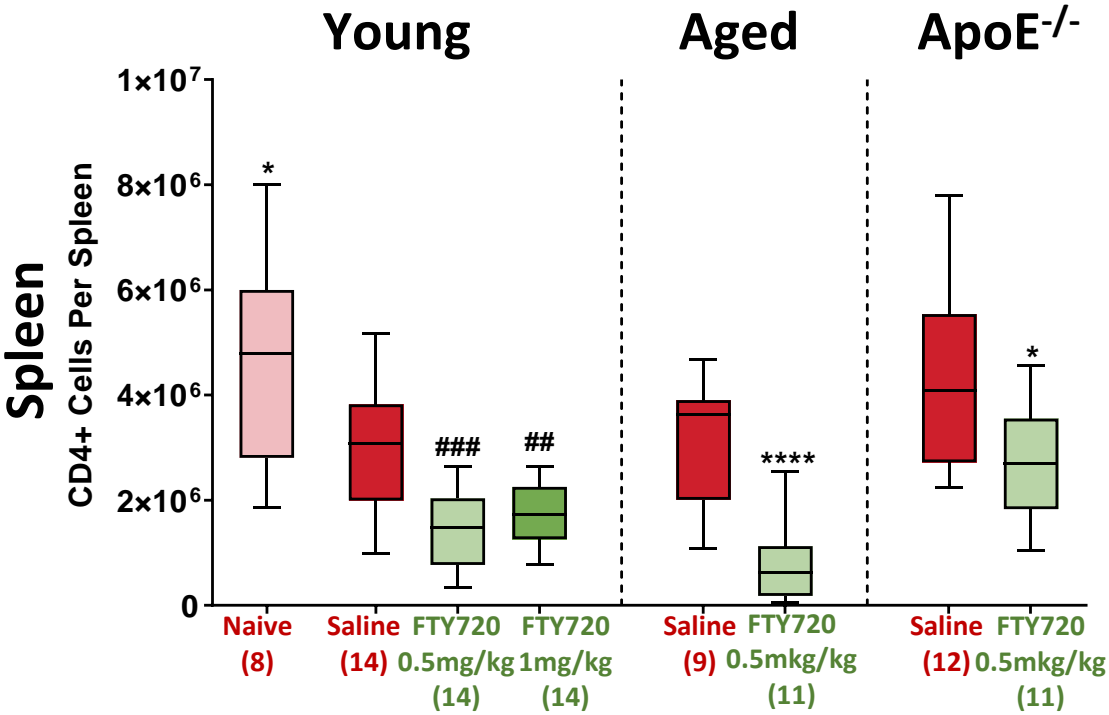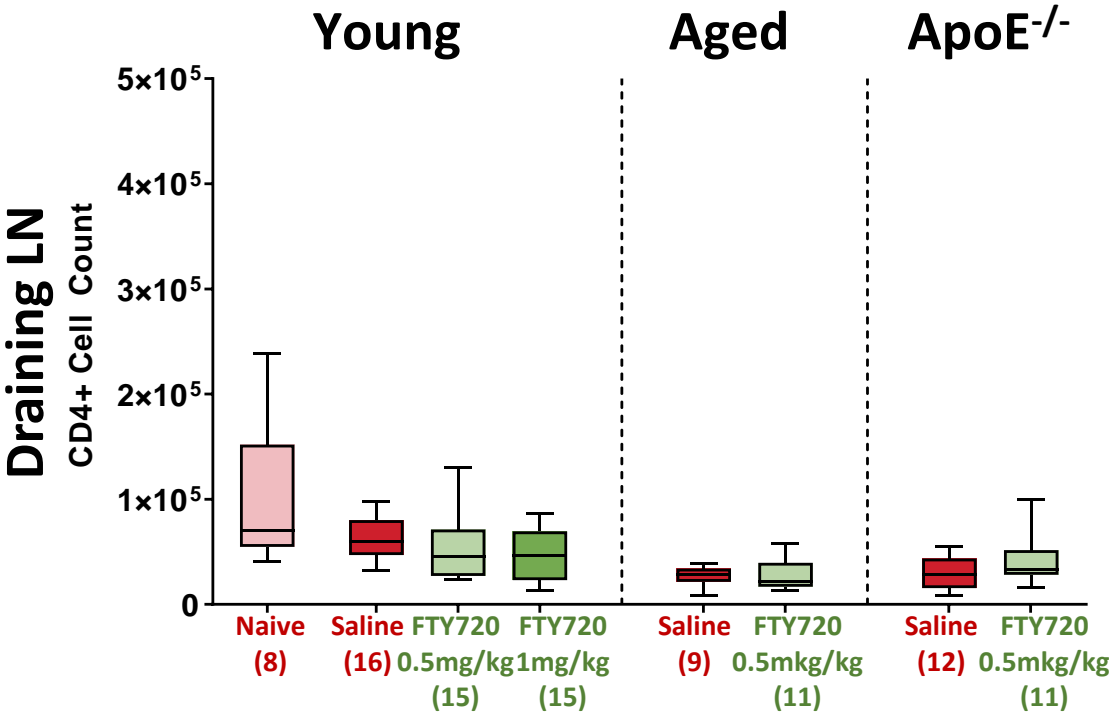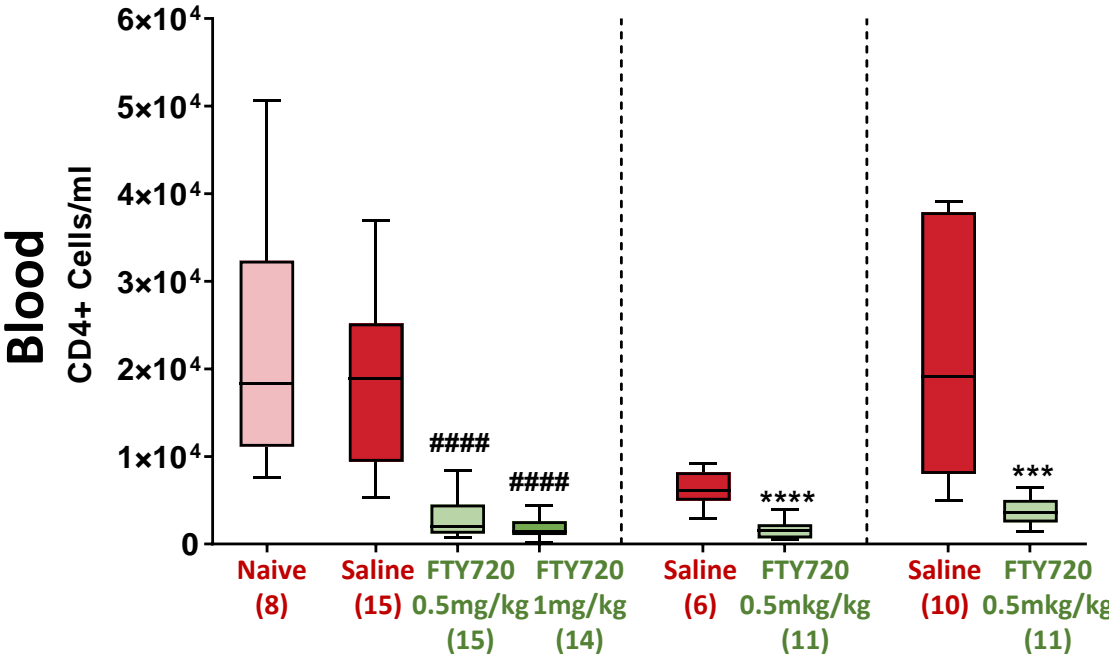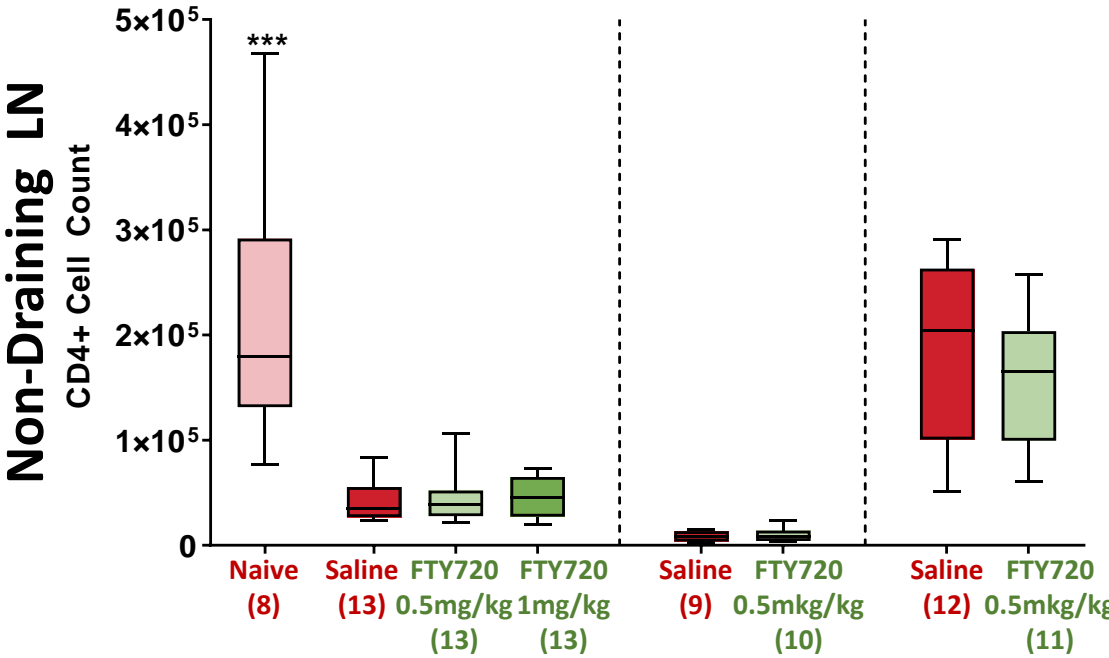

Supplement: Supplementary file 3 — Additional file 3. : Total CD4+ cell counts in blood and secondary lymphoid tissue across three individual mouse cohorts of brain ischaemia (t = 7 days). Two-sided, independent-samples t tests investigated differences between two groups (* = p<0.05, ** = p<0.01, *** = p<0.001 as compared to saline). One-way analysis of variance (ANOVA) tests with post hoc Tukey’s multiple comparisons were performed to investigate differences between three or more groups (# = p<0.05, ## = p<0.01, ### = p<0.001 as compared to saline). Number of mice per group shown in parentheses on x-axis.Box-and-whisker plots exhibit 10-90 percentiles. [file 12974_2021_2083_MOESM3_ESM.pdf]

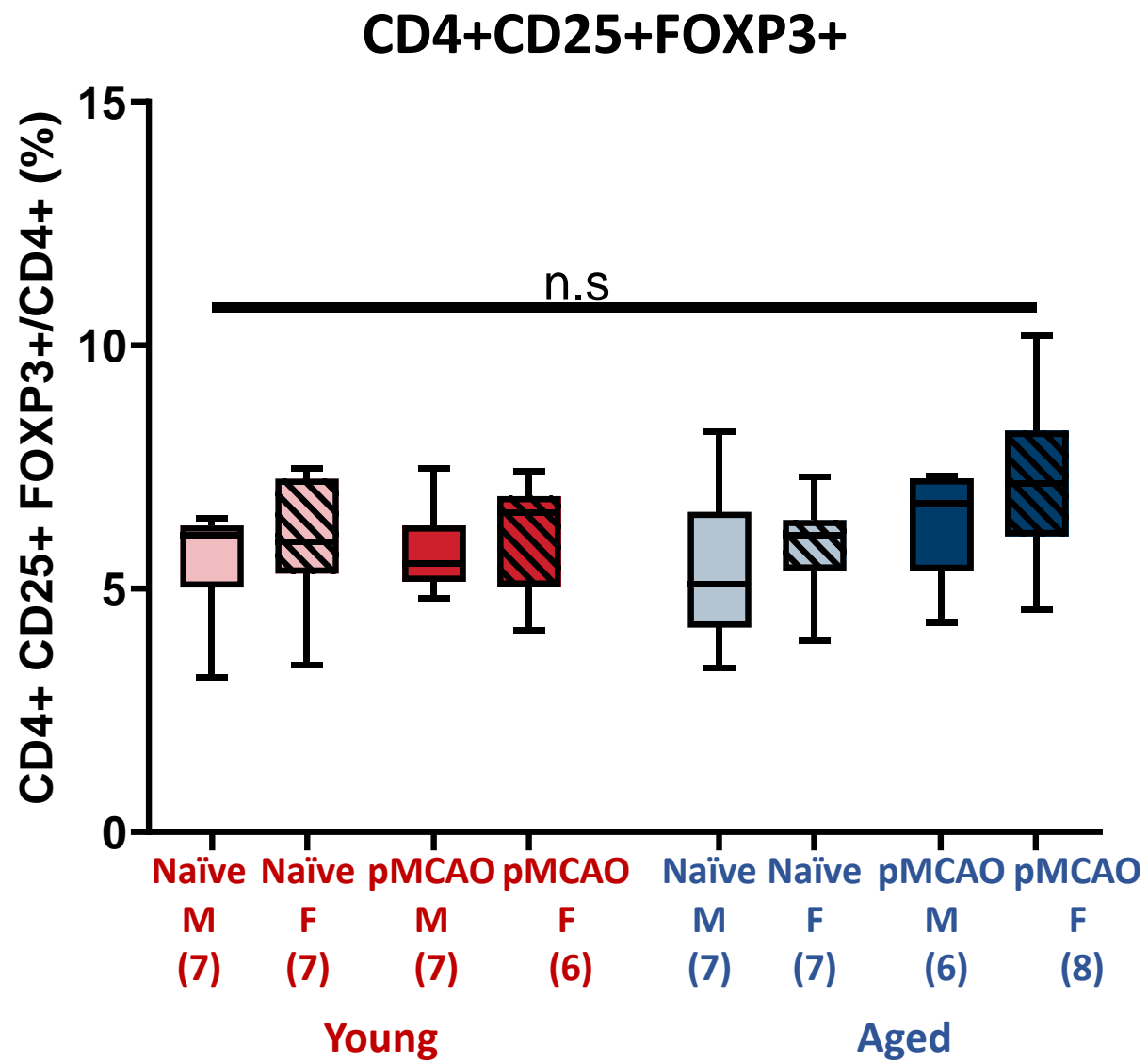

Supplement: Supplementary file 4 — Additional file 4. : Effect of pMCAO on the frequency of CD4+ CD25+ FoxP3+ cells in blood and secondary lymphoid tissue of young (male + female) and aged (male + female) mice (t = 7 days). Young mice are shown in red, aged mice are shown in blue. Male mice are shown in clear boxes, female mice are shown in hatched boxes. Naïve mice were age-matched mice who did not undergo any surgery. One-way analysis of variance (ANOVA) tests with post hoc Tukey’s multiple comparisons were performed to investigate differences between groups (# = p<0.05, ## = p<0.01, ### = p<0.001). The number of mice per group shown in parentheses on x-axis.Box-and-whisker plots exhibit 10-90 percentiles. [file 12974_2021_2083_MOESM4_ESM.pdf]
