# Supplementary material for: The effect of fingolimod on regulatory T cells in a mouse model of brain ischaemia
Source: J Neuroinflammation. 2021 Jan 30;18:37. doi: 10.1186/s12974-021-02083-5 (PMC7847573; doi:10.1186/s12974-021-02083-5)
Supplement: Supplementary file 5 — Additional file 5. : Detailed breakdown of number of mice excluded pre-surgery, mid-surgery for serious uncontrollable haemorrhage, or post-surgery for reaching a humane endpoint. Details of any naïve mice added, as well as samples missed or excluded post-analysis by ROUT method are also outlined. [file 12974_2021_2083_MOESM5_ESM.pdf]

Study	# of mice allocated to pMCAO/Sham	# of mice excluded for serious uncontrollable haemorrhage	# of mice culled for reaching humane endpoint (HEP)	# of surgical mice tissue collected from	# of samples excluded by ROUT method	# of samples included in final analysis
Dose response in young mice	51 (+ 8 naïve controls)	3	1	47 (except n = 44 spleen and n = 46 blood)	n = 0 cell frequency<br>n = 2 cell counts	n = 55 unless otherwise indicated
Aged	32	3	2	20	n = 0 cell frequency<br>n = 3 cell counts	n = 20 unless otherwise indicated
ApoE$^{-/-}$	38	7	0	23 (except n = 22 blood)	n = 0 cell frequency<br>n = 1 cell counts	n = 23 unless otherwise indicated
Young vs. Aged mice (including naïve)	39 (+28 naïve controls)	2	1	55	n = 0 cell frequency<br>n = 0 cell counts	n = 64 unless otherwise indicated
Treatment Duration	52 (+ 9 naïve controls)	2	2	45 (except n = 44 blood)	n = 0 cell frequency<br>n = 0 cell counts	n = 57 unless otherwise indicated
**Total:**	**257**	**17**	**6**	**190**	**n = 0 cell frequency**<br>**n = 6 cell counts**	**N = 219**
